# Development and Evaluation of a PSMA-Targeted Nanosystem Co-Packaging Docetaxel and Androgen Receptor siRNA for Castration-Resistant Prostate Cancer Treatment

**DOI:** 10.3390/pharmaceutics14050964

**Published:** 2022-04-29

**Authors:** Yingying Zhang, Hongxia Duan, Heming Zhao, Lingling Qi, Yanhong Liu, Zheao Zhang, Chao Liu, Liqing Chen, Mingji Jin, Youyan Guan, Zhonggao Gao, Wei Huang

**Affiliations:** 1 State Key Laboratory of Bioactive Substance and Function of Natural Medicines, Institute of Materia Medica, Chinese Academy of Medical Sciences and Peking Union Medical College, Beijing 100050, China; zyy@imm.ac.cn (Y.Z.); dhx@imm.ac.cn (H.D.); zhaoheming@imm.ac.cn (H.Z.); qilingling@imm.ac.cn (L.Q.); liuyanhong@imm.ac.cn (Y.L.); zhangzheao@imm.ac.cn (Z.Z.); chaoliu@imm.ac.cn (C.L.); chenliqing@imm.ac.cn (L.C.); jinmingji@imm.ac.cn (M.J.); 2 Beijing Key Laboratory of Drug Delivery Technology and Novel Formulations, Department of Pharmaceutics, Institute of Materia Medica, Chinese Academy of Medical Sciences and Peking Union Medical College, Beijing 100050, China; 3 Department of Urology, National Cancer Center/National Clinical Research Center for Cancer/Cancer Hospital, Chinese Academy of Medical Sciences and Peking Union Medical College, Beijing 100021, China

**Keywords:** prostate cancer, androgen receptor, siRNA delivery, docetaxel, PSMA targeting

## Abstract

Primary prostate cancer (PC) progresses to castration-resistant PC (CRPC) during androgen deprivation therapy (ADR) in early stages of prostate cancer. Thus, rather than blocking the androgen-related pathway further, docetaxel (DTX)-based therapy has become the most effective and standard first-line chemotherapy for CRPC. Although the therapy is successful in prolonging the survival of patients with CRPC, chemotherapy resistance develops due to the abnormal activation of the androgen receptor (AR) signaling pathway. Thus, to optimize DTX efficacy, continued maximum suppression of androgen levels and AR signaling is required. Here, we designed a prostate-specific membrane antigen (PSMA)-targeted nanosystem to carry both DTX and AR siRNA (Di-PP/AR-siRNA/DTX) for CRPC treatment. Specifically, DTX was encapsulated into the hydrophobic inner layer, and the AR siRNA was then condensed with the cationic PEI block in the hydrophilic outer layer of the PEI-PLGA polymeric micelles. The micelles were further coated with PSMA-targeted anionic polyethylene glycol-polyaspartic acid (Di-PEG-PLD). In vitro and in vivo results demonstrated that the resulting Di-PP/AR-siRNA/DTX exhibited prolonged blood circulation, selective targeting, and enhanced antitumor effects. Consequently, Di-PP/AR-siRNA/DTX holds great potential for efficient CRPC treatment by combining chemotherapy and siRNA silencing of androgen-related signaling pathways.

## 1. Introduction

Prostate cancer (PC) was the most common cancer diagnosed in men in the United States in 2021, accounting for 26% of the diagnoses and causing the second highest number of deaths in men [1]. Although a series of treatments for PC, including surgical castration, androgen deprivation therapy (ADT), and chemotherapy and radiation therapy [2], have been significantly refined and improved in recent years, the 5-year relative survival rates for PC by stage at diagnosis and race have reached 98% among the selected cancer types [1]. Repeated use of single therapeutic agents may lead to undesirable and severe adverse effects; for example, most patients have difficulty accepting surgical castration therapy [3,4]. Worse still, with the development of the disease, ADT causes almost all patients to become less sensitive to androgens and progress to castration-resistant prostate cancer (CRPC) [5]. Moreover, the treatment of patients with CRPC with cytotoxic chemotherapy has been proven to prolong the survival of patients to some extent. However, acquired drug resistance eventually occurs [6,7]. The major reason for chemotherapy resistance is the abnormal activation of the androgen receptor (AR) signaling pathway [8]. In addition, clinical data suggest that even if patients with PC enter the CRPC stage, the androgen signaling pathway still plays an important role in the progression of PC, and the antitumor efficacy of DTX in CRPC could be improved by inhibition of AR-pathway signaling [9,10]. Therefore, combination therapy of chemotherapeutic drugs and inhibitors of androgen-related signaling pathway inhibitor may effectively suppress CRPC.

The FDA has approved mitoxantrone, DTX, and cabazitaxel as treatment drugs for CRPC. Mitoxantrone was approved for CPRC in 1996; however, it has been clinically proven that the drug only relieves the pain of bone metastasis and cannot prolong the overall survival of patients [6]. Cabazitaxel was approved by the FDA in 2010 for patients with metastatic CRPC that progressed after DTX [7]. In 2004, DTX, a second-generation taxane family drug, was found to inhibit the mitosis and proliferation of cancer cells through multiple mechanisms, including promoting the assembly of microtubule dimers into microtubules, stabilizing the microtubules by preventing the demultimerization process, and blocking cells in the G2 and M phases. DTX has been proven to prolong the survival of patients with CRPC, and it is the only first-line chemotherapy drug approved by the FDA for advanced PC [11,12]. Since then, DTX-based chemotherapy has been widely used for the treatment of advanced PC. Before 2010, DTX was the first and only life-prolonging drug for PC treatment [13]. Therefore, DTX was chosen as the chemotherapeutic drug for this study owing to its superior efficacy.

The human AR gene is located at q11–12 of the X chromosome and contains eight exons and seven introns. The full length of the gene is approximately 90 kb, the molecular weight is approximately 110 kD, and it encodes 918 amino acids [14]. Under normal conditions, the AR has a regulatory effect on the transcription of downstream genes and assists in maintaining the normal structure and function of the prostate. In PC tissues, AR downstream target genes are over-transcribed, which promotes the proliferation and metastasis of PC cells. Simultaneously, the AR plays an important role in the occurrence and development of PC. Therefore, blocking the AR pathway is an important strategy for treating PC [15,16,17]. Moreover, compared with traditional therapies, the mechanism of siRNA therapy is clear and can be applied to all targets [18]. Inspired by their critical role in PC treatment and the optimal anti-tumor effects of the combination of chemotherapy and RNA interference, we speculate that the combined therapy of AR siRNA and DTX is likely to have an enhanced therapeutic effect on PC. However, DTX is a hydrophobic compound, whereas siRNA is a negatively charged and hydrophilic substance that is quickly degraded by nucleases. Therefore, the focus of this study is to achieve the targeted and coordinated treatment of these two drugs at the tumor site of CRPC.

Luckily, the availability of successful drug delivery carriers has opened up bright prospects for tackling the aforementioned issues. Recently, amphiphilic copolymers, especially those with hydrophobic segments to encapsulate hydrophobic compounds and hydrophilic positively charged shells to condense nucleic acids, have received great attention. Polyethylenemine (PEI), poly(dimethylaminoethyl methacrylate) (PDMAEMA), and poly(2-aminoethyl ethylene phosphate) (PPEEA) are the most frequently used cationic segments, and the most popular hydrophobic polymers are polylactide(PLA), poly(ε-caprolactone) (PCL), and poly(lactic-co-glycolic acid) (PLGA) [19]. However, it is widely accepted that the toxicity of cationic polymers remains a major obstacle in their application. Accordingly, modifications that reduce toxicity without compromising the gene delivery efficiency are required [20]. Another critical question that needs to be answered is how targeted drug delivery can be achieved. Prostate-specific membrane antigen (PSMA), which is highly expressed in normal and malignant prostate epithelial cells, is considered a tumor marker with higher specificity and sensitivity than prostate specific antigen (PSA). Therefore, PSMA is an attractive candidate for selective targeted therapy of prostate and other solid tumors. Saurabh Aggarwal et al. [21], from Johns Hopkins University, screened out linear 12 amino acid peptides with the sequence WQPDTAHHWATL from a random phage library that can selectively bind to PSMA. In addition, dimerization of this peptide resulted in enhanced binding to PSMA and an approximately 10-fold better inhibition of PSMA activity than the monomeric peptide. In addition, the selected dimeric peptide specifically bound to PSMA-producing PC cells with no significant binding to non-PSMA-producing cells.

**Figure 1:**
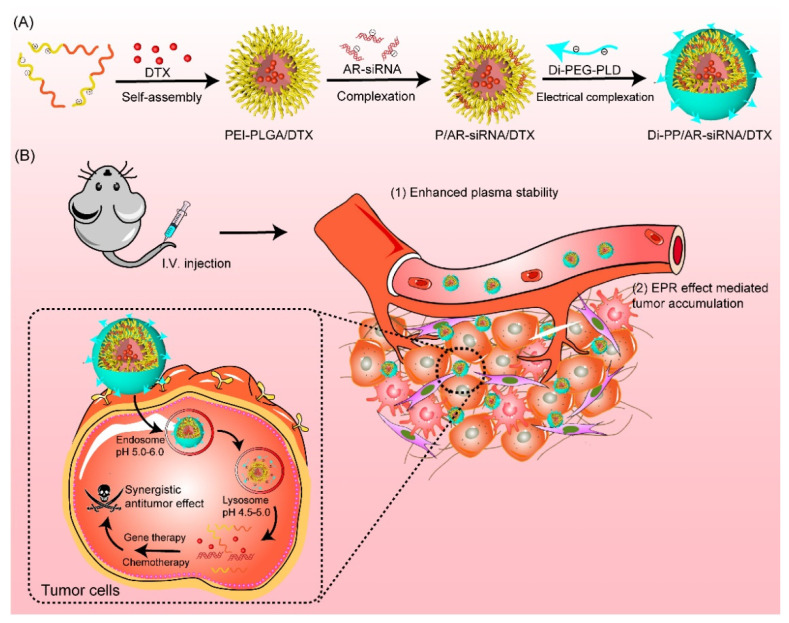
Illustration of the preparation and the mechanism of Di-PP/AR-siRNA/DTX. (**A**) The formation of Di-PP/AR-siRNA/DTX. (**B**) The mechanism of anti-tumor activity of DTX and AR siRNA.

Combining these ideas, we first designed a PSMA-targeted nanosystem to carry both DTX and AR siRNA (Di-PP/AR-siRNA/DTX) for CRPC treatment (Figure 1). In detail, DTX was readily encapsulated into the hydrophobic inner core of PEI-PLGA to obtain PEI-PLGA/DTX nanoparticles by ultrasonic emulsification with ice-water and solvent evaporation methods. The negatively charged hydrophilic siRNA was encapsulated in the cationic polymer PEI segment of the PEI-PLGA/DTX nanoparticles through electrostatic interactions. The synthesized dimeric peptide-linked polyethylene glycol-polyaspartic acid (Di-PEG-PLD) was then covered on the surface of PEI-PLGA/DTX/siRNA nanoparticles (P/AR-siRNA/DTX) to target PC. This final formed nanocomplex was named PEI-PLGA/DTX/siRNA/Di-PEG-PLD (Di-PP/AR-siRNA/DTX). When Di-PP/AR-siRNA/DTX reached the tumor site under the guidance of dimeric peptide, the nanocomplexes were first taken up by the tumor cells. Subsequently, the AR siRNA and DTX were rapidly released. The released AR siRNA silenced AR expression and effectively blocked the AR pathway, while DTX promoted the assembly of microtubule dimers into microtubules and stabilized the microtubules, thereby inhibiting mitosis and the proliferation of cancer cells. Thus, the two drugs have an enhanced anti-prostate cancer effect. Given its inclusiveness and tumor-specific drug delivery capacity, this study may provide a promising anti-PC therapy with a combination of nucleic acids and small-molecule chemotherapy drugs. 

## 2. Materials and Methods

### 2.1. Materials

Poly (lactic-co-glycolic acid) with carboxyl groups on one end (75/25, PLGA_2000_-COOH) was purchased from Jinan Daigang Technology Co (Shandong, China). Branched polyethyleneimine (MW = 1.8 kDa, bPEI_1__800_) was obtained from J&K Chemical Ltd. N-maleimide-PEG_5k_-PLD_10_ and Dipeptide (WQPDTAHHWATL) were commercially obtained from Tanshu-Tech (Guangzhou, China). Docetaxel was provided by Aladdin Reagent Database Inc. (Shanghai, China). Anti-androgen receptor siRNA (siRNA: 5′-AAGAAGGCCAGUUGUAUGG AC-3′) and Cy5-siRNA were synthesized by GenePharma Co., Ltd. (Shanghai, China). Ethidium bromide (EtBr) and 6-coumarin (C6, purity > 99%) were obtained from Sigma-Aldrich Inc. (Shanghai, China). Fetal bovine serum (FBS) and RPMI-1640 media were purchased from GIBCO, Invitrogen Corp. (Carlsbad, CA, USA). A cell counting kit-8 (CCK-8) was obtained from Dojindo Laboratories (Kumamoto, Japan). The primary antibody against androgen receptor used for WB and IHC was purchased from Abcam Ltd. (ab133273, Cambridge, UK). The fluorescein isothiocyanate (FITC)-annexin V/propidium iodide (PI) apoptosis detection kit was purchased from Beijing 4A Biotech Co., Ltd. Matrigel was bought from BD Biosciences (Bedford, MA, USA). The TUNEL detection Kit was purchased from Roche Ltd. (6432344001, Basel, Switzerland). All other chemicals were of analytical grade and used without further purification.

The LNCaP (a prostate cancer cell line) was acquired from the Department of Pathology in the Institute of Medicinal Biotechnology at Peking Union Medical College and cultured in RPMI 1640 medium supplemented with 10% FBS at 37 °C in a humidified atmosphere containing 5% CO_2_.

BALB/c nude mice (male, 6 weeks, 16−18 g) were purchased from Beijing Huafukang Biotechnology Co., Ltd. (Beijing, China) All animal experiments were approved by the Laboratory Animal Ethics Committee in the National Cancer Center/National Clinical Research Center for Cancer/Cancer Hospital in Peking Union Medical College. All experimental procedures conformed to institutional guidelines and protocols for the care and use of laboratory animals.

### 2.2. Synthesis and Characterization of PEI_1800_-PLGA_2000_ and Di-PEG_5k_-PLD_10_

PEI_1800_-PLGA_2000_ was synthesized as previously described [22,23,24]. Briefly, 100 mg PLGA_2000_-COOH, 115.1 mg NHS (molar ratio 1:20), and 191.5 mg EDC (molar ratio 1:20) were dissolved in an appropriate amount of DMSO and stirred at room temperature for 2 h. Then, 20 μL of triethylamine and 90 mg of PEI_1800_ (molar ratio 1:1) were added to the reaction mixtures and stirred for another 20 h at room temperature. The reaction mixture was dialyzed against deionized water in a molecular weight cut-off (MWCO) 2500 dialysis bag for 48 h to remove impurities and obtain PEI_1800_-PLGA_2000_. In addition, the final product was lyophilized. The structure of PEI_1800_-PLGA_2000_ was characterized using ^1^H NMR spectroscopy (600 MHz, Varian Medical Systems, Inc., Palo Alto, CA, USA) and further verified using Fourier transform infrared spectroscopy (Nicolet 5700; Thermo Fisher Scientific, Beijing, China).

A total of 20 mg Dipeptide and 69 mg Mal-PEG_5k_-PLD_10_ (molar ratio 1:2) were dissolved in 10.0 mL of HEPES buffer (0.1 M) at pH 8.0 and stirred at room temperature for 20 h under nitrogen protection. The resulting product was also purified by dialysis against deionized water with an MWCO of 2500 for 24 h. The solution was then lyophilized. The final resulting Di-PEG_5k_-PLD_10_ was characterized using ^1^H NMR and further confirmed using matrix-assisted laser desorption/ionization time-of-flight mass spectrometry (MALDI-TOF-MS) (4800 Plus, Applied Biosystems Inc., Waltham, MA, USA).

### 2.3. Preparation of DTX/siRNA-Loaded Nanodrug

PEI-PLGA/DTX nanoparticles (P/DTX) were prepared using the ice-water bath ultrasonic-emulsification vacuum solvent evaporation method. PEI-PLGA and DTX were dissolved in a mixed solvent consisting of 2 mL of dichloromethane and 2 mL of methanol and added dropwise to deionized water (the ratio of the aqueous phase to the organic phase was 9:1) under stirring. After the addition, the mixture was continuously stirred for 5 min to form a primary emulsion and sonicated with a probe-type sonicator at 150 W for 20 min. The formed colloid was rotary evaporated under reduced pressure at room temperature for 30 min to remove the organic solvent, passed through a 0.45 μm filter membrane, and lyophilized.

PEI-PLGA/DTX/siRNA nanoparticles (P/AR-siRNA/DTX) were prepared according to the mass ratio of PEI-PLGA to siRNA of 10, 20, 40, 80, 120, and 160. Specifically, PEI-PLGA/DTX was diluted with distilled water at various concentrations and mixed with an equal volume of siRNA solution, which was diluted with RNase-free water. The mixture was immediately vortexed for 30 s, followed by incubation at room temperature for 45 min. The PEI-PLGA/siRNA nanoparticle preparation method was the same as that for P/AR-siRNA/DTX, except for the change from PEI-PLGA/DTX to PEI-PLGA. Thereafter, the Di-PEG-PLD solution was mixed with P/AR-siRNA/DTX solution in equal volumes at different C/N ratios of 1:0.5, 1:1, 1:2.5, 1:5, 1:10, and 1:20, followed by incubation at room temperature for 45 min. The final solution was lyophilized to obtain PEI-PLGA/DTX/siRNA/Di-PEG-PLD (Di-PP/AR-siRNA/DTX, where “Di” refers to the targeted dimeric peptide, the first “P” refers to “PEG-PLD”, and the second “P” refers to “PEI-PLGA”). Moreover, the PEI-PLGA/DTX/siRNA/PEG-PLD (PP/AR-siRNA/DTX) nanocomplexes were prepared by mixing the P/AR-siRNA/DTX and PEG-PLD solution and further incubating for 45 min at room temperature.

The size and zeta potential of Di-PP/AR-siRNA/DTX were measured using a Malvern Zetasizer Nano ZS90 instrument (Malvern Instruments Ltd., Worcestershire, UK). The morphology was investigated by transmission electron microscopy (TEM, (Hitachi H-7650; Hitachi, Tokyo, Japan)). The siRNA-binding ability of the various formulations was investigated by agarose gel electrophoresis. Briefly, the siRNA-loaded nanodrugs prepared at different mass ratios of PEI-PLGA to siRNA and different C/N ratios of Di-PEG-PLD to PEI-PLGA were loaded onto a 4% agarose gel and electrophoresed at 150 V for 15 min in TAE buffer solution. After staining the agarose gel with 0.5 μg/mL ethidium bromide for 30 min, the retardation of the siRNA bands was visualized at 365 nm using a UV gel image system (SIM135A, SIMON). The quantity of DTX in the nanodrugs was detected using high-performance liquid phase (HPLC) (Agilent 1100 system, Santa Clara, CA, USA) equipped with a C18 column (5 μm particle size, 250 mm × 4.6 mm) with a mobile phase consisting of acetonitrile:distilled water = 65:35 (*v*/*v*) introduced at a flow rate of 1 mL/min. The detection wavelength was set to 230 nm. The entrapment efficiency (EE) and drug-loading (DL) were calculated, respectively, as follows:

EE (%) = (weight of DTX in nanodrug/feeding weight of DTX) × 100%,

DL (%) = (weight of DTX in nanodrug/weight of the nanodrug) × 100%

### 2.4. In Vitro Serum Stability and pH-Sensitive Release of DTX

Di-PP/AR-siRNA/DTX nanocomplexes were incubated at 37 °C in PBS supplemented with 10% FBS. The average size and zeta potential of the nanocomplexes were monitored by dynamic light scattering (DLS) over a period of 24 h.

The release kinetics of DTX from Di-PP/AR-siRNA/DTX nanocomplexes was evaluated through a dialysis method. Briefly, 0.5 mL solution containing 20 mg of Di-PP/AR-siRNA/DTX nanocomplexes was placed in a clamped dialysis bag (MWCO = 10,000 Da) and then immersed in 10 mL of buffer solutions (pH 7.4 or pH 5.5) containing 0.1% (*w*/*v*) Tween 80. The experiment was kept in a shaking incubator at 37 °C at 100 rpm. At predetermined time points (0, 0.25, 0.5, 1, 2, 4, 6, 8, 12, 24, 36, 48 h, and 60 h), 0.5 mL of release media was removed, and an equal volume of fresh release buffer was supplemented. After the dialysis was completed, the dialysis bag was cut and continued to vibrate for 2 h, which was used as the release amount of the drug for an infinite time. The DTX concentrations of different samples were determined by HPLC, as mentioned above.

### 2.5. Cellular Uptake Studies

Cy5-siRNA and C6 were used to replace AR-siRNA and DTX as model drugs, respectively. PEI-PLGA/Cy5-siRNA/Di-PEG-PLD (Di-PP/Cy5-siRNA), PEI-PLGA/C6/Di-PEG-PLD (Di-PP/C6), and PEI-PLGA/Cy5-siRNA/C6/Di-PEG-PLD (Di-PP/Cy5-siRNA/C6) nanocomplexes were prepared according to the above-mentioned preparation method. Qualitative cellular uptake efficiency of co-loaded nanocomplexes was assessed by confocal laser scanning microscopy (CLSM). LNCaP cells were separately seeded in confocal dishes (Nunc, Rochester, NY, USA) at a density of 2 × 10^5^ cells/well and incubated overnight. Cells were incubated with various formulations of nanocomplexes (5 μg/mL C6 and 100 nM siRNA) at 37 °C for 4 h. The medium was removed and discarded, and the cells were washed three times with cold PBS, followed by fixation with 4% paraformaldehyde for 15 min. DAPI was subsequently added to stain the nuclei. Fluorescent images of the cells were analyzed using a laser scanning confocal microscope (TCS SP2, Leica, Wetzlar, Germany).

To quantify cellular uptake efficiency, LNCaP cells were seeded into a 6-well plate and cultured overnight for attachment. The cells were then treated with Di-PP/Cy5-siRNA, Di-PP/C6, and Di-PP/Cy5-siRNA/C6, respectively (3 μg/mL C6 and 30 nM siRNA). After an incubation period of 4 h, the medium was removed, and cells were collected by centrifuged at 500× *g* at 4 °C for 5 min. Finally, the cells were resuspended in 500 µL of PBS for analysis using a FACSCalibur flow cytometer (Becton Dickinson, Franklin Lake, NJ, USA).

### 2.6. In Vitro Gene Silencing Efficiency Assay

Western blot analysis was used to evaluate the silencing effects of siRNA, Di-PP, Di-PP/NC.siRNA, and Di-PP/AR-siRNA on AR in LNCaP cells. LNCaP cells were seeded into a 6-well plate at a density of 2 × 10^5^ cells/cell and incubated in a humidified atmosphere of 5% CO_2_ at 37 °C for 24 h, after which the cells were cultured with various siRNA-loaded formulations at a concentration of 100 nM in the serum-free medium. After 4 h of incubation, the cells were washed three times with PBS and further incubated in complete medium for an additional 68 h. Subsequently, LNCaP cells in each group were lysed with RIPA buffer containing 1 mM phenylmethanesulfonyl fluoride. Protein samples (5 μg/μL) were electrophoresed on 10% sodium dodecyl sulfate–polyacrylamide gels. The proteins were then transferred onto a polyvinylidene fluoride (PVDF) membrane. After pre-incubating in blocking solution at room temperature for 1 h, the PVDF membrane was incubated with AR antibody overnight at 4 °C. After washing and further incubation with goat anti-rat immunoglobulin G (IgG) antibody for 40 min, bands were detected using an ECL system (ImageQuant LAS 4000 mini; Fuji, Tokyo, Japan).

### 2.7. In Vitro Cytotoxicity

First, the CCK-8 assay was used to evaluate the in vitro cytotoxicity of blank Di-PP. LNCaP cells were seeded in 96-well plates at a density of 8 × 10^3^ cells/well. After 24 h of incubation, the medium was replaced with Di-PP at a series of concentrations of 100, 10, 1, 0.1, and 0.01 μg/mL for 24 h. Next, CCK-8 was added and incubated for 3 h. The absorbance of the solution was recorded at 450 nm using a microplate reader (BioTek, Dallas, TX, USA). Each group comprised three samples in parallel, and the cell viability was calculated as follows:

Cell viability (%) = [(OD_sample_ − OD_blank_)/(OD_control_ − OD_blank_)] × 100%

For the anti-proliferation assay of the nanocomplexes, the cytotoxicity of Di-PP, Di-PP/NC.siRNA, Di-PP/AR-siRNA, Di-PP/NC.siRNA/DTX, and Di-PP/AR-siRNA/DTX on LNCaP cells was also evaluated using the CCK-8 assay, as described above. Briefly, after 24 h of incubation, the cells were cultured with the co-loaded samples for another 72 h and analyzed as described above. The concentrations of DTX and siRNA were 0.05 μg/mL and 12.5 nM, respectively.

### 2.8. Cell Apoptosis Assay

LNCaP cells were seeded into a 12-well plate at a population of 1.5 × 10^5^ cells/well and incubated in a humidified atmosphere of 5% CO_2_ at 37 °C. Twenty-four hours later, the cells were cultured with fresh medium, siRNA, DTX, Di-PP/AR-siRNA, Di-PP/NC.siRNA/DTX, PP/AR-siRNA/DTX, and Di-PP/AR-siRNA/DTX at a DTX concentration of 0.05 μg/mL and siRNA concentration of 12.5 nM for 72 h. After washing with PBS, the cells were fixed in 4% paraformaldehyde and stained with Hoechst 33258 (1 μg/mL) for 10 min, and apoptotic nuclei were observed using an inverted fluorescent microscope (IX51, Olympus, Tokyo, Japan). To quantify apoptotic cells, the treated cells were collected, stained with an Annexin V-FITC apoptosis detection kit, and immediately analyzed using flow cytometry.

### 2.9. The Expression of PSMA in Prostate Tissues

The subcutaneous tumor model was established by subcutaneous injection of a suspension of 2 × 10^7^ LNCaP cells in 200 μL of a mixture of PBS and Matrigel (1:1, *v*/*v*) into the forelimb of nude mice (male, six weeks age, 16−18 g of body weight). When subcutaneous tumors grew to approximately 80 mm^3^, the mice were euthanized, and their tumors were collected. The tumors were then fixed with 4% paraformaldehyde and cut into slices. Prostate histology was performed using PSMA staining.

### 2.10. In Vivo Tumor Growth Inhibition Study

The subcutaneous tumor model was established as described above. When subcutaneous tumors grew to approximately 70 mm^3^, the tumor-bearing mice were randomized into seven groups: saline, naked siRNA, Taxotere (DTX injection), Di-PP/AR-siRNA, Di-PP/DTX, PP/AR-siRNA/DTX, and Di-PP/AR-siRNA/DTX (*n* = 4). A combination of DTX (3 mg/kg) and siRNA (3 mg/kg) was given every three days for a total of five injections. Tumor volumes were measured (V = [major axis] × [minor axis]^2^/2), and body weights were recorded from the first day of treatment until the end of the experiment. On day 21, the mice were asphyxiated to death, and the main organs and blood samples were collected. All tumor tissues were photographed for qualitative comparisons and weighed for quantitative comparisons. In addition, apoptosis in tumor tissue was detected using the terminal-deoxynucleotidyl transferase mediated nick end labeling (TUNEL) assay using a TUNEL-POD kit according to the manufacturer’s protocol. Major organs (liver and lung) were collected, fixed with 4% PFA, and examined using paraffin H&E staining to study the potential pathological changes and compared with the similarly stained sections of organs from untreated healthy mice (*n* = 4).

### 2.11. Statistical Analysis

The results were all shown as mean values ± SD. The difference between two groups was considered significant when * *p* < 0.05, and very significant when ** *p* < 0.01 and *** *p* < 0.001. The *p* values were calculated according to the two-tailed Student’s t-test by SPSS 23 software (IBM Corporation; Armonk, NY, USA).

## 3. Results

### 3.1. Synthesis and Characterization of PEI_1800_-PLGA_2000_ and Di-PEG_5k_-PLD_10_

**Figure 2:**
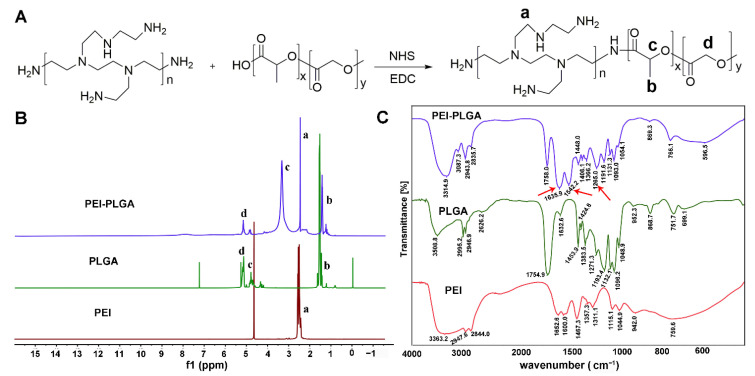
The synthesis and characterization of PEI_1800_-PLGA_2000_. (**A**) The synthetic scheme of PEI_1800_-PLGA_2000_. ^1^H NMR spectra (**B**) and FTIR spectra (**C**) of PEI_1800_-PLGA_2000_, PLGA, and PEI. RGD. a: –CH2-N of PEI; b: –CH3 of the PLGA block; c: –CH of the PLA block; d: –CH2 of the PLGA block. Red arrows show the characteristic absorption peaks of the amide bond: 1635.9 cm^−1^ (amide I), 1542.2 cm^−1^ (amide II), and 1265.0 cm^−1^ (amide III).

The synthesis of PEI_1800_-PLGA_2000_ was executed according to the scheme depicted in Figure 2A. As shown in Figure 2A, PEI_1800_-PLGA_2000_ was synthesized via the amidation reaction between the carboxyl group of PLGA-COOH and the amino groups of PEI. Figure 2B shows the ^1^H NMR spectra of PEI_1800_-PLGA_2000_, PLGA, and PEI, in which the proton signal peaks of 1.44, 3.34, and 5.13 ppm of PEI-PLGA correspond respectively to 1.54, 4.96, and 5.16 ppm in PLGA, which belong to the –CH3 of the PLGA block, –CH of the PLGA block and –CH2 of the PLGA block, respectively. In addition, the proton signal peak of 2.47 ppm of PEI-PLGA corresponds to 2.49 ppm in PEI, which belongs to the –CH2-N of PEI. The –CH in PLGA is connected to the carbonyl group, and the carbonyl group in PEI-PLGA forms an amide bond; thus, the –CH proton signal peak in PEI-PLGA was greatly shifted. All these spectroscopy results confirmed that PEI_1800_-PLGA_2000_ was successfully synthesized. In addition, as shown in Figure 2C, compared with PLGA, the infrared spectrum of PEI-PLGA showed the characteristic absorption peaks of the amide bond at 1635.9 cm^−1^, 1542.2 cm^−1^, and 1265.0 cm^−1^. FTIR results also showed that PLGA and PEI underwent an amide reaction to form PEI-PLGA.

**Figure 3:**
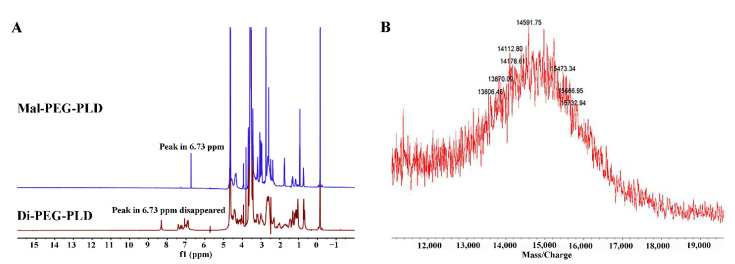
^1^H-NMR spectra of Mal-PEG_5k_-PLD_1__I0_ and Di-PEG_5k_-PLD_10_ (**A**) and MALDI-TOF-MS of Di-PEG_5k_-PLD_10_ (**B**).

Di-PEG_5k_-PLD_10_ was synthesized via the thiol-ene “click” reaction of the maleimide group of Mal-PEG_5k_-PLD_10_ with the sulfhydryl group of the dipeptide. Successful conjugation of Di-PEG_5k_-PLD_10_ was confirmed by the disappearance of the maleimide proton at 6.73 ppm compared to that of Mal-PEG_5k_-PLD_10_, as indicated in Figure 3A. The MALDI-TOF-MS result (Figure 3B) of Mal-PEG_5k_-PLD_10_ showed that the average MW of Di-PEG_5k_-PLD_10_ was 14,591.75 Da, which is consistent with the theoretical MW of Di-PEG_5k_-PLD_10_ (14,588.71), also confirming that the target molecule was successfully synthesized.

### 3.2. Preparation and Characteristics of DTX/siRNA-Loaded Nanodrug

**Figure 4:**
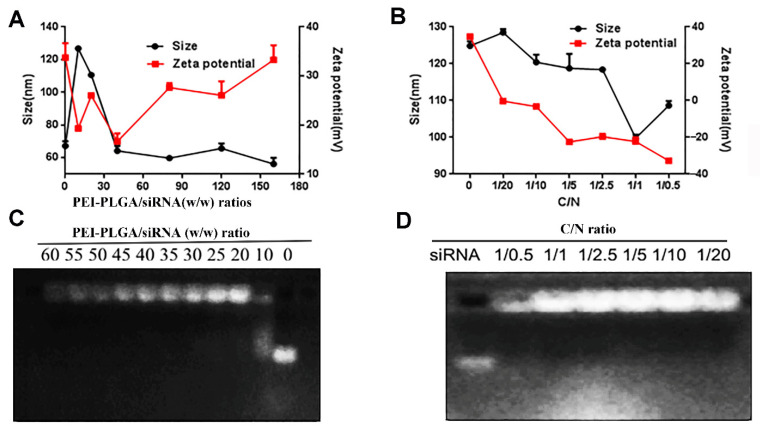
P/AR-siRNA/DTX and Di-PP/AR-siRNA/DTX NPs. (**A**) Particle size and zeta potential of P/AR-siRNA/DTX with various PEI-PLGA/siRNA (*w*/*w*) ratios (*n* = 3). (**B**) Particle size and zeta potential of Di-PP/AR-siRNA/DTX with Di-PEG-PLD coating at various C/N ratios (*n* = 3). (**C**) Gel electrophoresis assay for P/AR-siRNA/DTX with various PEI-PLGA/siRNA (*w*/*w*) ratios. (**D**) Gel electrophoresis assay for Di-PP/AR-siRNA/DTX with Di-PEG-PLD coating at various C/N ratios.

As shown in Figure 4A, when the mass ratios of PEI-PLGA to siRNA were 10, 20, 40, 80, 120, and 160, the particle size of P/AR-siRNA/DTX gradually decreased and tended to be smooth. When the mass ratio exceeded 40, the particle size stabilized below 100 nm. As the specific gravity of PEI-PLGA increased, that is, as the positive charge increased, the zeta potential of P/AR-siRNA/DTX continued to increase. Based on particle size and potential results, we believe that when the mass ratio of PEI-PLGA to siRNA was 40:1, the particle size and potential of P/AR-siRNA/DTX were more appropriate. The results of the agarose gel blocking method performed to examine the pyrolysis ability of PEI-PLGA with different mass ratios of siRNA are shown in Figure 4C. When the mass ratio of PEI-PLGA to siRNA was 40, PEI-PLGA could completely condense the siRNA.

The outer layer of P/AR-siRNA/DTX was coated with a layer of polypeptide-targeted anionic polymer Di-PEG-PLD via electrostatic interactions to obtain the nanocomplexes Di-PP/AR-siRNA/DTX. The particle size and zeta potential of the Di-PP/AR-siRNA/DTX nanocomplexes are shown in Figure 4B. With an increase in the C/N ratio, the zeta potential of Di-PP/AR-siRNA/DTX decreased, and the particle size decreased and then increased. This may be because a certain amount of negative charge is beneficial for stabilizing the positively charged P/AR-siRNA/DTX, but when the negative charge is excessive, the system becomes unstable. The results showed that when the C/N ratio was 1:1, the particle size was smallest (100 nm). Therefore, we selected a formulation with a C/N ratio of 1:1 as the preferred formulation. The results of gel electrophoresis in Figure 4D revealed that siRNA could be completely condensed at this ratio.

**Figure 5:**
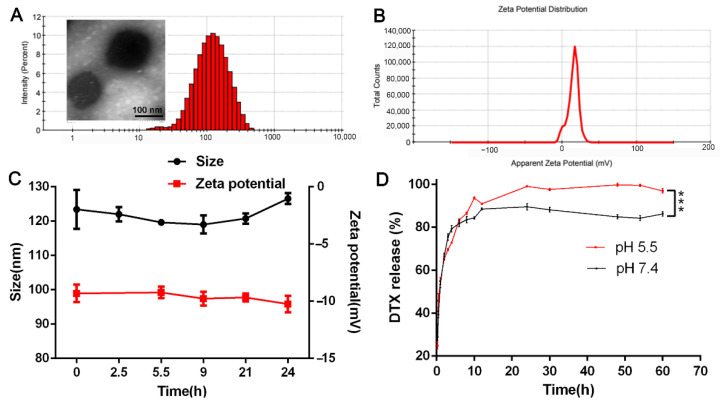
Characterization of Di-PP/AR-siRNA/DTX NPs. (**A**) Size and (**B**) zeta potential of Di-PP/AR-siRNA/DTX NPs. (**C**) The result of stability of Di-PP/AR-siRNA/DTX in PBS containing 10% FBS at 37 °C (*n* = 3). (**D**) DTX release profile of Di-PP/AR-siRNA/DTX at pH 5.5 and pH 7.4 (*n* = 3). *** *p* < 0.001.

Under the best formulation (the mass ratio of PEI-PLGA to DTX was 45:1, the mass ratio of PEI-PLGA to AR-siRNA was 40:1, and the ratio of Di-PEG-PLD to PEI-PLGA C/N was 1:1), the particle size of Di-PP/AR-siRNA/DTX was 101.4 nm, and the zeta potential was −20.1 mV (Figure 5A,B). TEM (Figure 5A) revealed that the Di-PP/AR-siRNA/DTX nanocomplexes were approximately in a regular spherical shape with a particle size of approximately 100 nm, which was mostly consistent with the particle size measured by DLS. The HPLC results showed that the EE% and LC% of DTX in P/AR-siRNA/DTX nanoparticles were 77.5% and 1.13%, respectively. In addition, the LC% of siRNA in P/AR-siRNA/DTX nanoparticles was 2.39%, and at this prescription, siRNA could be completely condensed.

As shown in Figure 5C, within 24 h, the particle size and zeta potential of Di-PP/AR-siRNA/DTX in PBS containing 10% serum did not change significantly. Moreover, we observed that Di-PP/AR-siRNA/DTX in PBS containing 10% serum still remained clear within 24 h. The solubility of free DTX in water is only 3.9 ± 0.2 µg/mL [25], so there would be obvious precipitation observed if there was DTX leakage. These findings suggested that Di-PP/AR-siRNA/DTX had good stability in plasma.

To investigate if the nanocomplexes could release DTX under pH stimuli, DTX release behaviors of nanocomplexes under pH 5.5 and pH 7.4 were measured, which simulated the slightly acidic tumor cell condition and blood circulation condition, respectively. Figure 5D shows that the cumulative release of DTX under pH5.5 conditions was significantly higher than under pH 7.4 conditions, especially after 8 h. This is because the polyaspartic acid on the outer layer of nanocomplexes is pH sensitive. Under the weakly acidic conditions of pH 5.5, the degree of dissociation of polyaspartic acid was low and changed into the neutral charge. The PEG-shell could not be tightly combined with the inner layer, and it detached. Therefore, the DTX contained in the inner layer was easy to release. These results can illustrate that the as-designed nanocarrier was pH-sensitive. As for the only 10% difference between the two groups, we think that it may because DTX was encapsulated in the innermost hydrophobic cavity and under the weakly acidic conditions of pH 5.5; only the PEG-shell was detached, but the DTX within the innermost hydrophobic cavity of PLGA-PEI micelles still presented sustained release. Perhaps the disassociation of the PLGA-PEI micelle is the rate-limiting step to control its release.

### 3.3. Cellular Uptake Studies

**Figure 6:**
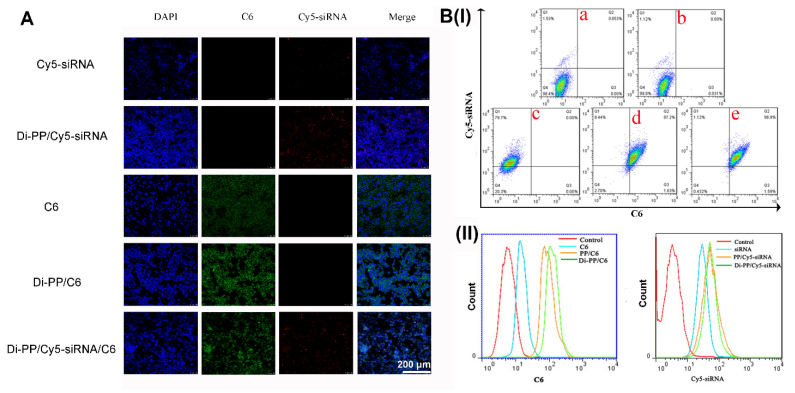
Intracellular distribution of various formulations that encapsulated C6 or/and Cy5-siRNA in LNCaP cells. Cellular uptake of C6 and Cy5-siRNA in different formulations by CLSM (**A**) and flow cytometry analysis (**B**). (**a**) negative control; (**b**) C6; (**c**) Cy5-siRNA; (**d**) PP/Cy5-siRNA/C6; (**e**) Di-PP/Cy5-siRNA/C6.

Higher cellular uptake and more specific intracellular distribution of DTX and AR-siRNA are vital for good therapeutic efficiency. The intracellular distribution of various formulations encapsulating C6 and Cy5-siRNA in LNCaP cells is shown in Figure 6. As illustrated in Figure 6A, the C6 and Cy5-siRNA that were not encapsulated by the carrier were rarely taken up by cells. After encapsulation into the nanoparticles, the cellular uptake ratios of C6 and Cy5-siRNA were significantly increased compared to the corresponding free dye. When nanoparticles co-loaded with C6 and Cy5-siRNA were given, green fluorescence of C6 and red fluorescence of Cy5-siRNA appeared at the same time, which proved that the synthesized carrier has the ability to simultaneously deliver hydrophobic chemicals and genetic drugs.

As shown in Figure 6B(I), compared with the negative control, free C6, and free Cy5-siRNA groups, the PP/Cy5-siRNA/C6 and Di-PP/Cy5-siRNA/C6 groups showed significantly increased cellular uptake of C6 and Cy5-siRNA. It is also obvious that the uptake of C6 in the PP/Cy5-siRNA/C6 and Di-PP/Cy5-siRNA/C6 groups increased from 0.031% to 87.2% and 96.9%, respectively, indicating that the delivery system used in the experiment played a significant role in promoting the endocytosis of drugs into cells. At the same time, the uptake rate of the Di-PP/Cy5-siRNA/C6 group was higher than that of the PP/Cy5-siRNA/C6 group, indicating the potent targeting ability of the dipeptide to LNCaP cells. In the uptake curves of C6 and Cy5-siRNA channels (Figure 6B(II)), PP/Cy5-siRNA/C6 and Di-PP/Cy5-siRNA/C6 groups could be seen to both have significant right shifts compared with the C6 group and siRNA, respectively. These results are consistent with those shown in the dual-channel quadrant graph.

### 3.4. In Vitro Gene Silencing Efficiency Assay

**Figure 7:**
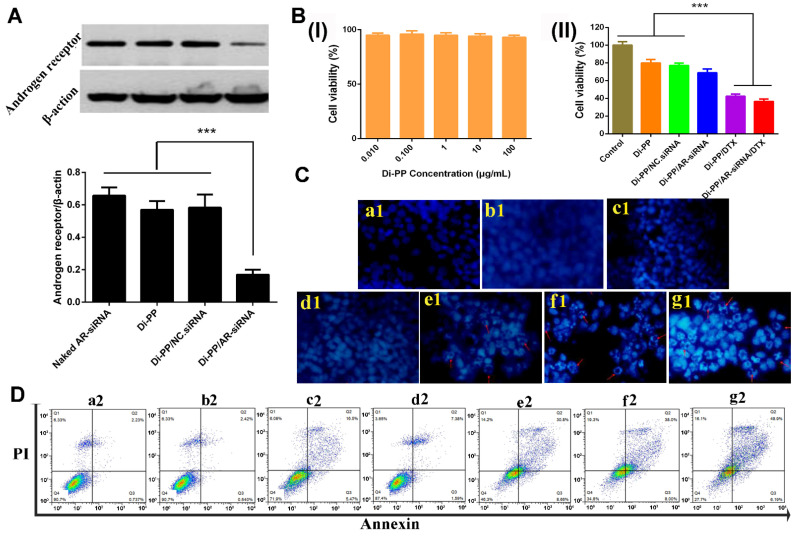
(**A**) Relative expression of androgen receptor protein levels in LNCaP cells after treatment with different formulations detected by Western blotting (*n* = 3) (*** *p* < 0.001). (**B**) (**I**) Cytotoxicity of Di-PP on LNCaP (*n* = 3). (**II**) Cell viability of LNCaP cells treated with different formulations for 72 h (*n* = 3) (*** *p* < 0.001). (**C**) Hoechst staining results. (**a1**) Negative control; (**b1**) siRNA; (**c1**) DTX; (**d1**) Di-PP/AR-siRNA; (**e1**) Di-PP/DTX; (**f1**) PP/AR-siRNA/DTX; (**g1**) Di-PP/AR-siRNA/DTX. Red arrows indicated apoptotic cells. (**D**) Annexin V-FITC/PI staining results. (**a2**) Negative control; (**b2**) siRNA; (**c2**) DTX; (**d2**) Di-PP/AR-siRNA; (**e2**) Di-PP/DTX; (**f2**) PP/AR-siRNA/DTX; (**g2**) Di-PP/AR-siRNA/DTX.

Androgen promotes the development of PC by binding to AR. AR-siRNA inhibits the expression of AR protein after uptake by PC cells, thereby preventing the binding of androgen and AR to inhibit the proliferation and metastasis of PC. In this experiment, the expressions of the AR protein in LNCaP cells after treatment with AR-siRNA, Di-PP, Di-PP/NC.siRNA, and Di-PP/AR-siRNA were analyzed by Western blotting. The result is shown in Figure 7A. Compared with the unencapsulated siRNA group (free AR-siRNA), blank nanoparticle group (Di-PP), and negative control nanoparticle group (Di-PP/NC.siRNA), the expression of the AR protein in the Di-PP/AR-siRNA group was significantly reduced, indicating that the designed delivery system could effectively encapsulate siRNA and deliver it into the cell to interfere with AR transcription and cause specific inhibition of the translation level [26,27].

### 3.5. In Vitro Cytotoxicity

As shown in Figure 7B(I), the cell viability of LNCaP cells was higher than 90% at the Di-PP concentration of 0.01, 0.1, 1, 10, and 100 μg/mL, indicating that the synthesized carrier was less toxic and safe.

The antiproliferative effects of different formulations on LNCaP cells after 72 h incubation are depicted in Figure 7B(II). The Di-PP/DTX group and Di-PP/AR-siRNA/DTX group showed comparable cytotoxicity and the strongest proliferation inhibition. However, the Di-PP/AR-siRNA group exhibited milder antiproliferative effects, with a cell viability of approximately 70%. The reason is that the AR-siRNA delivered into PCs could inhibit the expression of AR protein, thereby interfering with AR-dependent gene expression and inhibiting the proliferation and metastasis of PC cells, rather than directly inhibiting or killing tumor cells [28,29]. DTX can bind to microtubules to prevent mitosis and induce apoptosis [28]. Therefore, the toxic effect of the loaded AR-siRNA NPs on tumor cells was notably less significant than that of the cytotoxic chemotherapeutic agent DTX NPs.

### 3.6. Cell Apoptosis Assay

Cell apoptosis assays were performed to determine the antitumor efficacy of co-loaded AR-siRNA and DTX nanocomplexes. The Hoechst staining assay (Figure 7C) showed that in the negative control, naked siRNA, and DTX groups, the nuclei of all cells showed uniform blue coloration and relatively regular round shapes, and no obvious apoptotic cells were observed. The Di-PP/AR-siRNA group only showed a small number of apoptotic nuclei, whereas the Di-PP/DTX, PP/AR-siRNA/DTX, and Di-PP/AR-siRNA/DTX groups showed a large number of apoptotic cells with obvious nuclei fragmentation and irregular shapes. These results demonstrated that the DTX-loaded nanosystem had the ability to induce cell apoptosis.

**Figure 8:**
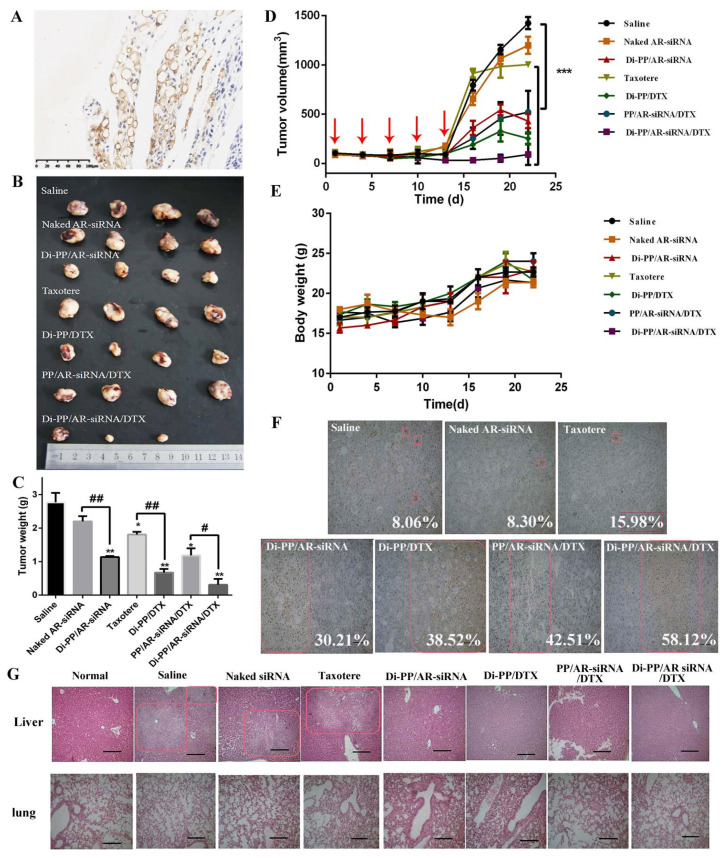
In vivo antitumor study of various formulations containing DTX and AR-siRNA in LNCaP tumor-bearing nude mice. (**A**) Immunohistochemistry image of tumor issues after stained with PSMA antibody, ×200. (positive cells shown in brown). The tumor photos (**B**), tumor weights (**C**), and the changes of tumor volume (**D**) and body weight of mice (**E**) (*n* = 4, # *p* < 0.05, ## *p* < 0.01). (**F**) TUNEL assay (representative 200× microscopy images) of tumor tissue from nude mice (scale bar = 50 μm). (**G**) H&E assay (representative 100× microscopy images) of lung and liver from nude mice (scale bar = 100 μm). * *p* < 0.05, ** *p* < 0.01, and *** *p* < 0.01.

The quantification of apoptotic cells was further investigated using the Annexin V-FITC/PI double-staining assay by flow cytometry, as depicted in Figure 8D. According to the manufacturer’s instruction of the used apoptosis kit, early apoptotic cells appeared in the lower right quadrant (Q3), and late apoptotic cells appeared in the upper right quadrant (Q2). The induced apoptosis of LNCaP cells by different formulations was characterized by counting the apoptotic percentages of the early and late periods (Q2 + Q3). In line with the results of the Hoechst staining assay, the total apoptosis rate of 9.0% and 38.8% induced by Di-PP/AR-siRNA and Di-PP/DTX, respectively, were higher than those induced by naked siRNA and DTX of 3.0% and 22.0%, respectively. Comparing the PP/AR-siRNA/DTX and Di-PP/AR-siRNA/DTX groups, the apoptotic rate of the target dipeptide group (55.1%) was higher than that of the non-target dipeptide group (46.0%).

### 3.7. The Expression of PSMA in Prostate Tissues

PSMA expression in LNCaP prostate tissues was detected using immunohistochemical staining (IHC). Positive expressions of PSMA were detected in LNCaP prostate tissue sections, as shown in Figure 8A. This further verified that subcutaneous inoculation of LNCaP cells in nude mice could form tumors with positive PSMA expression, which provides a basis for PSMA targeting in vivo and brings hope for achieving optimal effects of the drug [30,31].

### 3.8. In Vivo Tumor Growth Inhibition Study

During the administration period, the tumor growth rate in nude mice was slow, and tumor reduction was observed in the Di-PP/AR-siRNA/DTX group. After the therapy was stopped, the growth rate of the tumor increased, and the growth rate of the drug-loaded NPs group was slower, indicating the effectiveness of the designed drug delivery system (Figure 8B–D). The naked siRNA group showed no difference in tumor weight compared with the normal saline group. This may be because the naked siRNA was easily destroyed in the in vivo environment, making it difficult to exert its efficacy. Compared with the saline group, the tumor weights of the Taxotere, Di-PP/AR-siRNA, Di-PP/DTX, PP/AR-siRNA/DTX, and Di-PP/AR-siRNA/DTX groups were all significantly reduced (Figure 8C). Furthermore, the average tumor weights of mice receiving Di-PP/DTX and Di-PP/AR-siRNA were both noticeably lower than those of mice treated with the corresponding naked DTX and siRNA (*p* < 0.01). In addition, owing to the effective tumor targeting, mice treated with Di-PP/AR-siRNA/DTX displayed a higher rate of anti-tumor efficacy than PP/AR-siRNA/DTX (*p* < 0.05), as expected. Furthermore, no significant body weight loss was observed in any of the drug-loaded NP groups (Figure 8E), indicating no severe systemic toxicity of nanoparticles. 

After TUNEL staining, the nuclei of apoptotic cells were brown, and the nuclei of non-apoptotic cells were blue [32]. In order to explain the tumor inhibitory effect from the viewpoint of cells, we further quantitatively analyzed the percentage of apoptotic cells in each group. As shown in Figure 8F, there were few apoptotic cells in the saline (8.06%), naked siRNA (8.30%), and Taxotere groups (15.98%), while there were significant increases in the TUNEL-positive cells by the as-prepared NPs (Di-PP/DTX: 38.52%, Di-PP/AR-siRNA: 30.21%, PP/AR-siRNA/DTX: 42.51%, Di-PP/AR-siRNA/DTX: 58.12%). These results indicated that the enhanced anticancer effect of the as-prepared nanocomplexes was the result of inducing cell apoptosis.

H&E staining was performed on lung and liver tissue sections of nude mice to further observe the metastasis of PC. The results are shown in Figure 8G. The morphology of the lung tissue in each group was normal, and no obvious tumor metastasis was observed. Compared with normal nude mice, the liver tissue cells of the saline, naked siRNA, and Taxotere groups showed a nested arrangement, with large and deeply stained nuclei, as well as cell nuclei division. There were visible and small white spots on the liver of nude mice, indicating that PC had undergone liver metastasis. However, the liver tissues of nude mice in the single-loaded siRNA, single-loaded DTX, and dual-loaded drug non-targeting and targeted groups were similar to normal nude mice, and no obvious tumor metastases were observed, indicating that the drug encapsulated by the carrier had an inhibitory effect on the metastasis of PC.

## 4. Discussion

In general, as the most common cancer diagnosed and the second deadliest cancer in men in 2021, great efforts still need to be exerted to fight against PC. The occurrence and development of PC is a heterogeneity process involving a variety of signal molecules, signal pathways, and complex tumor microenvironments [33]. From this perspective, it is difficult to defeat this problematic disease with a single therapeutic agent, and combination therapy may be an intelligent strategy. As is well known, DTX-based therapy is still the standard first-line chemotherapy against CRPC. However, chemotherapy resistance quickly appears due to the abnormal activation of the AR signaling pathway. Therefore, the addition of DTX and abiraterone, an androgen biosynthesis inhibitor, has become clinically widely adopted [8,9,10,11,12]. However, compared with abiraterone, the mechanism of siRNA therapy is clear, and the mechanism can directly be applied to the AR target [19]. Thus, inspired by the clinical trials and attempting to provide a reference for clinical application, we designed a PSMA-targeted nanosystem to simultaneously carry DTX and AR siRNA (Di-PP/AR-siRNA/DTX) for CRPC treatment.

In vitro and in vivo experiments both showed that the as-designed Di-PP/AR-siRNA/DTX nanosystem was an effective and safe nanotherapeutic strategy. The detailed experimental results and reasoning can be clarified by the following aspects. Firstly, PEI is the most commonly used cationic materials and can been seen as the gold standard for loading generic drugs. However, the greater toxicity of PEI has always been problematic [34]. In this study, we linked the PEI with PLGA to form PLGA-PEI, which reduced the toxicity of PEI but did not affect its ability to carry generic drugs [35]. In this way, DTX could be encapsulated into the hydrophobic inner layer, while the AR siRNA could be condensed with the cationic PEI block in the hydrophilic outer layer of PEI-PLGA polymeric micelles, achieving co-loading drugs in one drug delivery system but with a different position. The way our system encapsulates drugs enables drugs to be well and more encapsulated, which is the premise to exert efficacy. Unlike many drug delivery systems against PCs at present, combination therapy requires that the two chemotherapeutic drugs, which are both hydrophobic, be packaged into the same hydrophobic cavity. The two are crowded with each other, leading to limited drug loading and failure to meet the effective dosage [36,37,38]. Moreover, the effective dose of siRNA itself is much lower than that of chemotherapeutic drugs [19], so this further reduces the pressure on our designed delivery system to contain drugs.

Secondly, the micelles containing AR-siRNA and DTX were further coated with PSMA-targeted anionic polyethylene glycol-polyaspartic acid (Di-PEG-PLD). On the one hand, this PEG modification improved the stability of the nanosystem during long blood circulation. On the other hand, Di-PEG-PLD, a kind of anionic polymer whose aspartic acid moiety is negatively charged at pH 7.4 but neutral at pH below 6.0, would detach from the nanosystem and hence avoid the “PEG dilemma” and give rise to rapid and triggered release of AR-siRNA and DTX [39]. It is due to good drug loading ability, high stability, and triggered release that the as-designed Di-PP/AR-siRNA/DTX nanosystem obtained higher cytotoxicity against LNCaP cells in vitro (Figure 7B(II),C,D), significantly decreased AR protein expression (Figure 7A), and obvious tumor inhibition in vivo (Figure 8B–D).

Thirdly, we should keep in mind at all times that another extremely important aspect for nanotherapeutics is their safety in addition to effectiveness. Compared with some delivery carriers studied for PC treatment at present, such as mesoporous silica nanoparticles [40], the materials we used, including PLGA and PLD, are biodegradable. Therefore, safety has been guaranteed (Figure 7B(I) and Figure 8E).

## 5. Conclusions

In this study, we first prepared Di-PP/AR-siRNA/DTX nanocomplexes co-loaded with DTX and AR-siRNA for the targeted and coordinated treatment of PC. The nanocomplexes improved the poor solubility of chemical drugs and enhanced the stability of gene drugs in vivo. In addition, as-designed Di-PP/AR-siRNA/DTX nanocomplexes effectively and selectively carried both DTX and AR-siRNA to PC tissues and cells, which further effectively downregulated AR expression and increased the therapeutic efficacy of DTX in PC. As a result, a potential anti-CRPC effect was gained. Overall, this PSMA-targeted nanosystem may provide a promising solution for the clinically-targeted treatment of PC using chemotherapeutic drugs and siRNA drugs that silence androgen-related signaling pathways.

## Data Availability

All data available are reported in the article.
